# Effect of long-term low dose prednisolone administration on bone mineral density: Relating to non-compliant women with rheumatoid arthritis

**DOI:** 10.22088/cjim.9.2.171

**Published:** 2018

**Authors:** Behzad Heidari, Parnaz Heidari, Karimollah Hajian-Tilaki, Mohammad Ali Bayani, Mansour Babaei

**Affiliations:** 1 Mobility Impairment Research Center, Health Research Institute, Health Research Institute, Babol University of Medical Sciences, Babol, Iran; 2 Clinical Research Development Unit of Ayatollah Rouhani Hospital, Babol University of Medical Sciences, Babol, Iran; 3 Tehran Islamic Azad University, Faculty of Medicine, Tehran, Iran; 4 Social Determinants of Health Research Center, Health Research Institute, Babol University of Medical Sciences, Babol, Iran

**Keywords:** Rheumatoid arthritis, Compliance steroid users, Prednisolone, Osteoporosis

## Abstract

**Background:**

Long-term treatment of rheumatoid arthritis (RA) with prednisolone (PRED) is associated with bone mineral density (BMD) loss. This study aimed to determine the status of BMD in non-compliant women who used PRED alone for RA.

**Methods:**

Non-adherent RA taking < 7.5 mg daily PRED without DMARDs for > 6 months, and RA patients taking methotrexate +PRED (RA control) were compared with age-matched non-RA controls. BMD was measured by dual energy x-ray absorptiometry (DXA) method and osteoporosis (OP) was diagnosed by WHO criteria. Influence of PRED on RA bone mass, and the risk of OP in RA was assessed by comparing PRED users RA and RA control,versus non-RA controls.

**Results:**

Sixty-four PRED user RA, 39 RA controls and 111 non RA-controls, with respective mean (±SD) age of 52±11; 8, 51±11; and 52±7.5 years (P=0.91) were studied. Median duration of treatment in PRED users and RA control was 2.5 and 4 years, respectively. BMD g/cm^2 ^at the femoral neck (FN-BMD) and lumber spine (LS-BMD) in PRED users and RA control was significantly lower than non-RA control (P=0.001). The prevalence of OP at either FN or LS in both RA groups was significantly higher than controls (P=0.001). In PRED users, the risk of OP increased by OR=4.9, P=0.001) and in RA controls by OR=1.7 (P=0.20). The risk of OP in PRED user RA was 2.89 times (P=0.014) greater than RA controls.

**Conclusions:**

These findings indicate significantly lower BMD, and higher prevalence of osteoporosis in non-compliant women with RA taking low-dose PRED alone for a median period of 2.5 years, as compared with patients taking standard treatment comprising methotrexate +PRED.

Rheumatoid arthritis (RA) is an inflammatory arthritis associated with bone loss and osteoporosis. Bone loss arises as a consequence of inflammatory process, impaired physical activity and inadequate treatment (1-4). The magnitude of bone loss varies according to severity of the disease and extent of suppression of inflammatory process with appropriate treatment using disease-modifying anti-rheumatic drugs (DMARDs) (5). Currently, treatment of RA emphasizes on early and regular use of anti-inflammatory drugs particularly low dose methotrexate (MTX) with or without low-dose prednisolone (PRED) directing to decrease disease activity and remission achievement (6). Adherence to prescribed medications and following treatment regimen has a major role in improvement and outcome (7, 8). Non-adherence to prescribed medications is expected to be associated with continuous disease activity and greater disability leading to greater bone loss. 

The percentage of RA patients following prescribed medications varies across different study populations (9, 10). In a randomly selected sample of 228 patients with RA, 32-40% of patients did not adhere to their DMARD prescription (8)11. In one study consisted of 788 randomly selected RA, only 64.3% of patients adhered to MTX. In this study, DAS28 score was significantly greater in the group treated with MTX in single therapy as compared with the group who were treated with glucocorticoid alone (10). In one study of 108 RA, 39% of the patients were non-compliant with their treatment regimen varying from occasional to complete failure to take prescribed medications (12).

In RA patients, adherence rate to oral MTX and steroid ranges from 58-71% (12). Among the non-compliant RA patients, adherence to take low-dose PRED seems to be greater than other anti-inflammatory drugs due to its rapid symptomatic action on joint pain and tenderness (13). Non-adherent steroid user RA patients are at greater risk of bone loss and osteoporosis as compared to adherent RA patients who follow conventional treatment containing MTX or other DMARDs in combination with PRED. As glucocorticoids exert some disease-modifying effect on bone due to its anti-inflammatory effect, and thus may partly compensate adverse effects (13). Nevertheless, glucocorticoid therapy is independently associated with increased risk of bone mineral density (BMD) loss in the hip and spine (14). In chronic users, cumulative dose of glucocorticoid causes further bone loss and increases the risk of osteoporosis at least two times (15). Despite several published studies regarding complications of long-term glucocorticoids therapy in RA, nevertheless BMD status in non-compliant steroid user RA has not been investigated yet. This study was performed to determine the influence of low-dose PRED in non-adherent RA patients who used low-dose steroids as the sole drug for treatment of disease without any DMARDs for prolonged period. 

## Methods

The patients of this case- control study were selected consecutively according to inclusion criteria among RA the patients who referred to the outpatient rheumatology clinic in Babol for treatment evaluation over a one-year- period in Babol, Iran. The diagnosis of RA was confirmed by the 1987 American College of Rheumatology revised criteria (16). Data were provided with respect to adherence to prescribed medications, type of medications and duration of treatment via a face-to-face interview. Non-adherent patients who used low-dose PRED (< 7.5 mg/day) without DMARDs longer than 6 months as the sole drug for treatment of RA were considered as steroid user group (RA cases) and adherent RA who followed the standard treatment including MTX with or without low-dose PRED, were considered as control (RA control). 

Exclusion criteria included psoriatic arthritis, systemic lupus erythematous, undifferentiated arthritis, and the presence of coexistent conditions leading to altered bone metabolism. A group of age-matched non-RA patients were also selected as non-RA controls among women presented to the same clinic for bone densitometry. A similar exclusion criterion was also applied for the control group. BMD gr/cm^2 ^was assessed in the femoral neck (FN) and lumbar spine (LS) regions by dual x-ray absorptiometry (DXA). Sample size was estimated to detect a 20% difference between the patients and the control group. Based on our earlier study (6), thus 30 patients for each group would be adequate to detect such difference with power of 80% and confidence level of 95%. However, we included further patients to compensate the missing data. The results of bone densitometry were compared with the reference data supplied by the manufacturer and expressed as BMD g /cm^2^, BMD T-score, BMD Z-score for each measurement site. The proposal of this study was approved by the Ethics Committee of Babol University of Medical Sciences, Babol, Iran. The primary objective of this study was to determine the influence of long-term low-dose PRED on RA bone mass by comparison of steroid user RA and RA controls with age-matched non -RA control as regards mean BMD g /cm^2 ^differences and frequency of osteoporosis. The secondary objective was to determine the risk of osteoporosis in each group of RA by calculating odds ratio (OR) with corresponding 95% confidence interval (95% CI). Diagnosis of osteoporosis was confirmed by the WHO criteria (17) (BMD-T -score less than - 2.5) and the frequency of osteoporosis was determined at either FN or LS based on the lowest T -score (T-score < 2.5) of the measured skeletal sites as proposed by the International Society for Clinical Densitometry (one diagnostic category) (18). Distribution of all variables was determined by Kolmogorov- Smirnov test. Nonparametric Mann-Whitney U test and independent t test was used for comparison of quantitative variables with skewed or normal distributions, respectively. 

## Results

Sixty-four steroid user RA patients with mean (±SD) age of 52±11.8 years, 39 RA controls with mean (±SD) age of 51±11 years, and 111 non-RA controls with mean (±SD) age of 52±7.5 years (P=0.91) were studied. Median disease duration (DD) in steroid users and RA controls was 6 (0.16-40) and 9 (1-21) years (P=0.001) and median duration of treatment was 2.5 (0.12-20) and 4(0.16-10) years, respectively (P=0.001) (table 1). The two groups of RA were compared with regard to percentage of RF (P=0.55) and prevalence of anti-cyclic citrullinated peptide antibody positivity (P=0.35). FN- BMD g /cm^2 ^and LS- BMD g /cm^2 ^in both RA groups were significantly lower than non-RA controls (P=0.001 for both).The BMD g /cm^2 ^at both sites, in steroid user RA was significantly lower than RA controls (P=0.001) (table 1). Compared with non-RA controls, The BMD at FN, in steroid users was lower than RA controls (data not shown) (24.3% vs 16%, P=0.74). Corresponding values for LS- BMD g /cm^2 ^between steroid users and RA controls as compared with non-RA controls were 12.9% and 8.4% (data not shown) respectively, none (P= 0.29). 

Osteoporosis at either FN or LS was observed in 36 (56,2%) in steroid user patients versus 12 (30.8%) patients in RA control group (0.001). In logistic regression analysis after adjustment for menopausal duration, compared to non-RA control, the risk of osteoporosis at either FN or LS increased by odds of 4.90 (95% CI, 2.50-9.65. P=0.091) in steroid users RA and 1.7(95%CI, 0.75-3.86, P=0.20) in RA controls (table 1). Risk of osteoporosis at FN or LS in steroid user RA was 2.89 times greater than RA controls (OR=2.89.95% CI, 1.24-6.7, P=0.014).

**Table 1:** Characteristics of women rheumatoid arthritis (RA) who were treated with long-term low-dose prednisolone monotherapy (steroid users), or conventional drugs including combination of low-dose methotrexate and low-dose prednisolone compared with age-matched non-RA controls

**Characteristics of patients**	**Standard therapy** ≠ **(RA controls)**	**Steroid users (RA cases)** ҂	**Non-RA Controls**	**P-value**
No	39	64	111	
Age (yrs)	50.6±11.4	52.5±11.8	52±7.5	0.91
Disease duration (years) median( range)	9 (1-22)	6 (0.16-40)	-	0.001
Treatment duration (years) Medan (range)	4 (0.16-10)	2.5 (0.12-20)	0.001	0.001
RF positivity, no (%)^α^	27 (69.8)	44 (69.2)		0.55
Anti-CCP positivity, no(%)^β^	30 (76.9)	50 (82)		0.35
Methotrexate, no (%)	39 (100)	0 (0%)		0.001
Low-dose prednisolone, no (%)	39 (100)	42 (67.7)		0.001
OP	12 (30.8)	35 (54.7)	23 (20.7)	
FN-BMD^¥^	0.73±0.10	0.68±.13	0.87±0.16	0.001
LS-BMD^¥^	0.85±0.15	0.81±0.15	0.93±0.16.	
Osteoporosis at either FN or LS^α^	12 (30.8)	36 (56.25)	23 (20.7)	0.001
Risk of osteoporosis ^€^	1.7 (0.75-3.8)	4.9 (2.5-9.6)	1	

≠ : RA patients treated with combination of low-dose prednisolone and methotrexate

҂ : RA patients treated with low-dose prednisolone alone <7.5 mg/daily for prolonged period

Α : Rheumatoid factor

β : Anti-cyclic citrullinated peptide antibody

¥ Femoral neck (FN) and lumbar spine (LS) bone mineral density (BMD)

€ Risk of osteoporosis at either FN or LS by logistic regression analysis

## Discussion

The results of this study indicated that patients with RA have lower BMD g /cm^2 ^compared with age-matched non -RA controls. Non-compliantt patients with RA who used low-dose PRED as the sole drug therapy for a median duration of 2.5 years had significantly lower bone mass as compared with age-matched controls, as well as with RA patients, who followed combination of low-dose PRED and MTX for a median duration of 4 years. In this study, the BMD in RA control, in spite of the longer disease duration than steroid user RA was higher, which should be attributed to synergetic effect of MTX and low-dose PRED (19). 

Compared with non -RA controls, in both RA groups, the mean BMD difference at the FN was higher than lumbar spine. This issue indicate that, in RA, bone loss at the femoral neck is greater than lumbar spine, which should be attributed to synovitis of hip (14).

The impact of low -dose PRED with or without MTX on RA bone mass has been addressed in several studies (20-25). Current data indicate that prolonged corticosteroid therapy is associated with high prevalence rate of osteoporosis (23, 24). Combination of steroids and DMARDs exerts beneficial effect in preserving greater bone mass (21, 26, 28-30). The findings of this study are in contrast with a few previous studies which have found no beneficial effect of MTX alone or in combination with low-dose PRED in preserving BMD (8, 31, 32). Nonetheless, duration of treatment in these studies was shorter than the present study. 

A systematic review of randomized controlled trials or cross- over trials demonstrated that, the addition of low dose PRED to the standard therapy can substantially reduce the rate of bone erosion in RA, or reduce bone erosions or disease progression (32). Nevertheless, with regard to bone mass status, the outcome of RA patients on long-term immunotherapy with low -dose PRED has not been compared with standard therapy including low- dose PRED and MTX. Sambrouk et al. in a study of 84 RA patients, found a significant BMD loss at FN and LS regions in both PRED-users and non-users RA .Percentage of BMD loss over an average period of 89.6 months did not differ between the two groups and the authors concluded that low dose oral corticosteroids did not increase the risk of generalized osteoporosis in patients with rheumatoid arthritis (33). 

The beneficial effects of corticosteroids in RA arise with decreasing synovitis, bone erosion, joint pain and increasing physical activities. In one study, RA patients who failed to stop PRED due to recurrence of joint pain, after two years, had no greater bone loss compared with those who discontinued , but in contrast, the value of BMD Z score in these patients was greater (20). The benefits of low- dose PRED in compensating the negative effect of inflammation on bone mass is clearer in early RA, which exerts a bone mass preserving effect particularly in the hip joint (24, 34). In addition, combination of low-dose PRED and DMARDs prevent or retard progression of radiographic erosions (26). The extent of contribution of steroid therapy in the development of osteoporosis in non-compliant RA is difficult to determine. Since, not only the inflammatory process, but other associated factors of osteoporosis may also cause low bone mass in RA (35-37). Furthermore, several factors like long disease duration, low to moderate disease activity and liver disease are associated factors of osteoporosis in RA, these factors themselves may decrease patient's compliance (38). 

Patients with RA are required to take multiple drugs for prolonged period of time. While self-efficacy encourages patients to follow treatment (26), fear of drugs toxicity is an important factor of non-compliance to treatment (39). In one prospective study of RA, over three years, 35.7% of patients were consistently compliant and 23.8% were persistently non-compliant to treatment. Older age, female sex and disability, significantly increased compliance (40) in addition, drug dosage, age of onset, affect the levels of adherence. In one study, the rates of compliance with treatment varied from 73% for etanercept, and 59% for MTX, to 6% for glucocorticoid (41) in a similar study, two years retention rate of MTX for psoriatic arthritis and RA were 65% and 66%, respectively (40). By lengthening of duration treatment, the rate of adherence to MTX in RA decreased from 68.2% in one year to 49.8% two years after the newly diagnosed elderly RA (19).

A significant benefit of MTX plus low-dose PRED on FN bone mass in RA should be attributed to anti-inflammatory effect of corticosteroid against hip synovitis. Synovial inflammation of hip joint has a major contribution in the development of radiologic damage and osteoporosis in FN region (14).

The findings of this study should be considered with limitations. In this study, the data regarding disease activity score were not provided before and after the treatment. Disease activity correlates with bone loss (14, 30). Furthermore, information concerning other conventional risk factors of osteoporosis (35, 36, 37) have not been collected. Yet, these factors are expected to be distributed similarly across the comparison groups, so the results might be confound minimally. Factors such as magnitude of BMD changes with treatment of RA, age at onset of RA may also affect the results (42, 43). Additionally, coexistence of RA with osteoarthritis falsely increases BMD, particularly at the lumbar spine and may cause underestimation of osteoporosis (44). The distribution of osteoarthritis is expected to be similar across the study groups and the results with minimal confounding effect.

In conclusion, this study indicates that non-compliant RA who used only low-dose PRED for treatment of RA, has significantly lower BMD and higher prevalence of osteoporosis, as well as greater risk of osteoporosis as compared with RA patients on conventional treatment comprising MTX and PRED. These findings indicate that low-dose PRED in combination with MTX exerts BMD preserving effect.
